# Evaluation of the Modulatory Effect of *Annona muricata* Extracts on the Activity of Some Selected Antibiotics against Biofilm-Forming MRSA

**DOI:** 10.1155/2021/9342110

**Published:** 2021-12-20

**Authors:** David Neglo, Clement Okraku Tettey, Edward Ken Essuman, Justice Dziedzorm Amenu, Felix Charles Mills-Robertson, Daniel Sedohia, Adjoa Agyemang Boakye, Daniel A. Abaye

**Affiliations:** ^1^Department of Basic Sciences, School of Basic and Biomedical Sciences, University of Health and Allied Sciences, PMB 31, Ho, Volta Region, Ghana; ^2^Department of Biochemistry and Biotechnology, Kwame Nkrumah University of Science and Technology, University Post, Kumasi, AR, Ghana; ^3^Department of Biomedical Sciences, School of Basic and Biomedical Sciences, University of Health and Allied Sciences, PMB 31, Ho, Volta Region, Ghana; ^4^Department of Nutrition and Dietetics, School of Allied Health Sciences, University of Health and Allied Sciences, PMB 31, Ho, Volta Region, Ghana; ^5^Department of Biological, Physical and Mathematical Sciences, School of Natural and Environmental Sciences, University of Environment and Sustainable Development, PMB, Somanya, Eastern Region, Ghana; ^6^Department of Clinical Microbiology, Kwame Nkrumah University of Science and Technology, University Post, Kumasi, Ashanti Region, Ghana

## Abstract

The study investigated the influence of *Annona muricata* extracts on the action of selected antibiotics against biofilm-forming MRSA. The various parts of the plant were processed into powder and extracted with ethanol or hot water and then screened for the presence of phytochemicals. The modulatory effect of the *Annona muricata* extract was also tested on some antibiotics against Methicillin-resistant *Staphylococcus aureus* (MRSA). The findings from this study revealed that the various parts of the *Annona muricata* extract (ethanolic and aqueous) contained different proportions of secondary metabolites. Varied antimicrobial activities were observed when the extract of the *A. muricata* was exposed to MRSA strain at a concentration of 100 mg/mL. The stem recorded the highest (17.00 and 18.00 mm) inhibitory activity against MRSA for both the aqueous and the ethanolic extract, respectively, and this was not different from the control, tetracycline. Again, the results on the modulatory action indicated that out of the 10 extracts of *A. muricata*, 4 of them antagonized the activity of ampicillin against the tested MRSA by a factor of 0.5 folds and the rest potentiated the drug within 1–4 folds, respectively. On the other hand, the various test extracts significantly potentiated the efficacy of streptomycin and tetracycline against the MRSA by a range of 1–32 folds with the aqueous root extract recording the highest synergistic effect and ethanol seed extract with the least effect. The findings of this study support the antibacterial activities of the *A. muricata* plant parts.

## 1. Introduction

Contemporarily, a notable number of noncommunicable and infectious diseases have become an indispensable headache in many countries, accounting for the majority of the increase of death tolls in most tropical nation states, especially Africa [1, 2]. Undoubtedly, this ever persistence of these deadly illnesses can be attributed to the modern-day problem of drug resistance caused by pathogens in place of unselective use of over-the-counter drugs [3] as well as factors that include failure to complete a prescribed dosage, irrational drug use, prolonged drug use, and therapy duration [4].

In effect, besides the latest discovery of resistance mechanisms through scientific researches such as efflux pump possession, target site diversion, resistance gene acquisition, and others by bacterial species to inactivate the potency of most antibacterial agents [5, 6], bacterial biofilms have also been reported and noted to form 65% of microbial infections, and bacteria living in them develop resistance to antibiotics a thousand times than those existing as planktonic cells, thereby calling for urgent attention [7]. This, therefore, has made the treatment of infections difficult, whereas surgical and other medical procedures have also been contemporarily associated with high-risk interventions causing prolonged sickness, disability, and death [8]. Consequently, the capacity to use phytochemicals to modulate the activities of commercial antimicrobials to overcome resistance from pathogens has developed through research [4, 9]. Hence, research is being carried out in recent times into the synergism of conventional antibiotics to establish and develop new drugs to tackle the antimicrobial resistance menace.

*A. muricata* is commonly known as soursop and one of the most important traditional medicinal plants which contain a lot of chemicals that exhibit various pharmacological properties [10]. It has since been adopted in traditional medicine to treat varied diseased conditions ranging from fever to diabetes and cancer. Over 200 bioactive compounds have been secluded from this plant, some of which are alkaloids, phenols, and acetogenins [11]. This plant is extensively disseminated in the tropical regions of Central and South America, Western Africa, Central and Eastern Africa, and Southeast Asia [11].

Recent work on the phytochemistry of *A. muricata* extracts has proven as a potent antioxidant, antimicrobial, anti-inflammatory, insecticidal, larvicidal, and cytotoxic against cancer cells. Other studies have also proven them as anxiolytic, antistress, anti-inflammatory, immunomodulatory, antimalarial, antidepressant, gastroprotective, wound healing, hepatoprotective, hypoglycemic, anticancer, and antitumoral agents [12, 13]. The commonest use of its preparations in traditional medicine is the hot water preparation of the bark, root, seed, or leaves. Moreover, recorded in the Brazilian Amazon, the leaves of the plant are used to treat liver problems, and the extracted leaf oil has been proven to help cure rheumatism, neuralgia, and arthritis. It is worth noting that some tropical sub-Saharan countries involving Uganda have been testified to employ all parts of this plant to treat malaria, stomachache, parasitic infections, diabetes, and cancer [14]. Its fruit juice has been used to cure diarrhoea, heart, and liver diseases [12, 15], and it is effective against intestinal pathogens in South America. This study intended to explore the influence of *Annona muricata* extracts on the actions of selected antibiotics against biofilm-forming Methicillin-resistant *Staphylococcus aureus* (MRSA).

## 2. Materials and Methods

### 2.1. Source of Samples, Identification, and Preparation

The selected parts of *Annona muricata,* namely, roots, stem bark, leaves, fruit, peels, and seeds, were harvested from a house in Ho Municipality after undergoing identification and authentication by the Institute of Traditional and Alternative Medicine, University of Health and Allied Sciences (UHAS), Ghana (Table 1). The voucher specimens were later deposited at the herbarium of the above-mentioned institution. The earth materials of these plant materials as well as undesirable materials were washed under a running tap, sliced into smaller pieces, and air-dried under shade for 7 days for leaves and 21 days for the whole stems and roots. Each dried plant part was converted into a coarse powder using an electric mill and then subjected to extraction with the appropriate solvent.

### 2.2. Extraction Procedure

The method of extraction was done according to the procedure described by Mills-Robertson et al. [16] with slight modifications where 70% ethanolic and hot water extractions (decoctions) were implemented to showcase the traditional style of administration of medicine. During the ethanolic extraction, 300 g of each of the coarse powders of the various above-mentioned parts with 200 g of peels and seeds was cold macerated in a proportion of 1 : 10 (W/V) of 70% ethanol at room temperature for a day. Likewise, for the hot water extraction, 300 g of each of the course powders of the test samples with 200 g of each of the peels and seeds was soaked in the various proportions of distilled water as stated above for 30 min and immediately subjected to boiling for 30 min. These preparations were then left to simmer at 60°C for 30 min and further cool at room temperature. Subsequently, the extracted solutions were filtered out, concentrated by employing the use of Buchi Rotavapor (R-200), and then freeze-dried. These portions were stored at 4°C in the refrigerator until use.

### 2.3. Phytochemical Screening

The presence of alkaloids, flavonoids, steroids, terpenoids, saponins, tannins, and glycosides was tested per the method described by Visweswari et al. [17] with slight modification. Grading of the final reaction of the secondary metabolites was done by comparing the results obtained for the plant extracts using how deep or light the colour change was.  Table 2 shows a summary of the various tests that were conducted.

### 2.4. Test Organism

The test MRSA strain, NCTC 12493, used in this study was provided by the Biomedical Science Department (microbiology unit), University of Health and Allied Sciences, Ho, Ghana, and standardized to 0.5 McFarland for use.

#### 2.4.1. Quantification of Biofilms Formation of the MRSA

The biofilm-forming potentials of the standard MRSA diluted to 0.5 McFarland standard in Mueller Hinton broth were employed per the protocols described by Elhadidy and Elsayyad [18] and Stepanović et al. [19] with slight modification. Briefly, 200 *µ*L of the standardized inoculum prepared was added to 2 mL of broth in test tubes and incubated at 37°C for 24 h.

Planktonic MRSA cells were then aspirated and washed from the tubes to get rid of floating cells, subsequently dried at 25–28°C in an incubator, and stained with 2 mL of 0.1% crystal violet for 15 min after which they were washed with sterile water and dried at room temperature. The adherent MRSA biofilms on the walls of the tubes were then reconstituted with 2 mL ethanol, and the optical density of each test sample was read at 595 nm with a UV spectrophotometer (Jenway, Bibby Scientific Ltd, Stone, Staff., UK) after being blanked. The optical density (OD) of the sterile broth was subtracted from that of the MRSA biofilms formed to exclude the background absorbance. Each of the procedures was performed in triplicate. These OD values were considered as an index of bacteria adhering to the surface and forming biofilms. The tubes were examined, and the amount of biofilm formation was scored as follows: 0 = nonbiofilm formation, 1+ = moderate biofilm formation, and 2+ = strong biofilm formation. Nonbiofilm formation with OD595 is <0.5, moderate biofilm formation with OD 570 ranges from 0.5 to 2.5, and strong biofilm formation with OD570 is >2.5. Uninoculated tubes containing Mueller Hinton broth (2 mL) served as the negative control. The data were then averaged, and the standard deviation was calculated.

### 2.5. Antimicrobial Susceptibility Testing

The agar well diffusion method was carried out per the protocol adopted by Irshad et al. [20] with slight modification. Approximately 20–25 mL of molten Muller Hinton Agar was distributed into petri dishes and allowed to [R] set. With the help of sterile swab sticks, each of the MRSA strains at a final concentration of 1.8 × 108 CFU/mL was acquired and was applied to the agar plates. Seven wells were created in each agar plate using a cork borer (No. 3, 5 mm). Into these holes, 50 *µ*L of 100 mg/mL of each test plant sample prepared with either sterile distilled water or 20% DMSO was introduced. Sterile autoclaved water or 20% DMSO was designated as negative control while 10 *µ*g of tetracycline/ketoconazole was taken as a positive control.

#### 2.5.1. Minimum Inhibitory Concentration (MIC) and Minimum Bactericidal Concentration (MBC)

The MICs of the crude extracts and reference drugs (ampicillin, tetracycline, and streptomycin) against MRSA were determined with the micro broth dilution method using 96-well microtiter plates per the protocol adopted by Eloff [21] and Ayensu and Quartey [22] with slight modification. The original stock concentrations of 128 mg/ml and 256 *µ*g/mL of each extract and antibiotics, respectively, were used to prepare well concentrations ranging from 32 to 0.25 mg/mL and 128 to 0.13 *µ*g/mL. The sterile 96-well plates were then prepared by pipetting into each well 100 *μ*L of double strength Mueller Hinton broth, 80 *μ*L of varied concentrations of test extracts or antibiotics, and 20 *μ*L of inoculums of a standardized suspension of MRSA to make a final volume of 200 *μ*L. The plates were then subjected to incubation at 37°C for 24 h, after which the activity in each well was perceived with MTT. Briefly, 20 *μ*L of MTT was added to each of the wells, and the results were noted after 30 min. Each experiment was conducted in triplicate. Subsequently, the minimum bactericidal concentration of each extract was deduced from the wells with the lowest concentrations having no colour change at which no growth occurs after subculturing for 24 h of incubation for MRSA carried out by Nester et al. [23].

### 2.6. Resistance Modulatory Studies of the Extracts

In this assay, 10 different concentrations of the wells prepared from 1000 mg/mL of stock solution of each extract of the plant parts were used as subinhibitory concentrations in conjunction with the antibiotics such that the concentrations arrived at were beneath the MICs of the antibiotics. Each well concentration was prepared such that a sole well of 200 *μ*L contained 0.5 mg/mL of each of the extracts used. Synchronously, the concentrations within the range of 0.13–128 ug/mL of the test antibiotics were prepared from their stock solutions. The well plates were prepared by dispensing into each well 100 *μ*L of double strength nutrient broth, 80 *μ*L of the test plant samples (each plant extract and antibiotics), and 20 *μ*L of the test standardized suspensions (10^6^ cells) of the test MRSA culture. The last column well (No. 12) served as a positive control, and the stocked broth in the test tube served as a negative control. Each of the labelled plates was then subjected to incubation at 37^o^C for 24 h. This was followed by the addition of 20 *μ*L of 3-(4, 5- dimethylthiazole-2-yl)-2,5-diphenyltetrazolium bromide (MTT) (0.1% w/v) to each well and incubated at 37°C for 30 min. The concentrations at which there was no colour change of the MTT added from yellow to purple compared to the next well concentration were noted as MICs.

### 2.7. Statistical Analysis

All measurements were expressed as mean ± SD of independent experiments using Microsoft Excel. Each of the tests was done in triplicate, and the average was recorded. Tukey's HSD test was conducted for results of the test that were statistically significant between groups and were determined by one-way ANOVA.

## 3. Results

### 3.1. Phytochemical Screening

The results in Table 3 showed the presence of saponins, terpenoids, phenols, and tannins in different proportions in the ethanolic and aqueous extracts of the various parts of *A. muricata* except in the aqueous extracts of the seed. Alkaloids were as well moderately noted in all different types of the extracts of the various parts except the aqueous extracts of the leaf, peel, and stem as well as noted in the ethanol peel extract. There was an absence of steroids in the various extracts of *A. muricata* except being temperately observed in the aqueous and ethanolic extracts of the root.

### 3.2. Biofilm-Forming Capacity

The results in Table 4 indicate that the MRSA produced a moderate amount of biofilm which may be partly responsible for its resistance.

### 3.3. Antimicrobial Activity Determination

**Figure 1:**
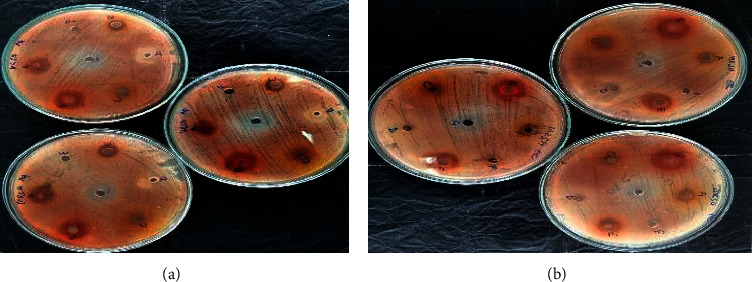
Aqueous (a) and ethanolic (b) extract of *A. muricata* against MRSA with respect to the plant parts: A: root extract; B: stem bark extract; C: leaf extract; D: seed extract; E: peel extract; F: water/20% DMSO; G: tetracycline/ketoconazole; Aq: aqueous; Eth: ethanolic.

Each of the test extracts of *A. muricata* demonstrated varied antimicrobial potential against MRSA at a concentration of 100 mg/mL as shown in Figure 1 and Table 5. The stem recorded the highest inhibitory activity on MRSA for both the aqueous and the ethanolic extract, whereas the aqueous seed extract, as well as the ethanolic peel extract, demonstrated the lowest inhibitory effects.

### 3.4. Minimum Inhibitory Concentration

The aqueous and ethanol extracts of the plant parts tested demonstrated antimicrobial activity against the pathogenic MRSA using the broth microdilution assay. Minimum inhibitory concentrations (MICs) of the plant extracts ranged from 0.25 to 16 mg/mL (Table 6). The lesser the MIC, the better the potency of the extracts. Among the 10 extracts tested against MRSA, the ethanol root extract demonstrated the lowest MIC potential of 0.25 mg/mL, comparable to others showing varied inhibitory potential as seen in Table 6. Additionally, the results on the minimum bactericidal concentration established that each of the different extracts was bacteriostatic at varied concentrations as shown in Table 6.

### 3.5. Antibiotic Modulatory Activity of Various Extracts of *A. muricata*

From the results obtained in Table 7, it was evident that out of the 10 extracts of *A. muricata*, 4 of them (aqueous extract of seed, root, peels, and stem) antagonized the activity of ampicillin against the tested MRSA by a factor of 0.5 folds and the rest potentiating the drug within 1–4 folds. Furthermore, the various test extracts significantly potentiated the efficacy of streptomycin and tetracycline against the MRSA by a range of 1–32 folds with the aqueous root extract recording the highest synergistic effect and ethanol seed extract with the least effect.

## 4. Discussion

The various parts of *Annona muricata* have been known for their ethnomedicinal properties due to the antimicrobial activity it possesses. The leaves and seeds have been extensively studied due to their traditional use. The phytochemical screening of the extract of the various parts of the plant together showed the existence of secondary metabolites, and this agrees with a previous report [24, 25]. The presence of these secondary metabolites makes pharmacological studies imperative. Over 200 bioactive compounds have been reported to be found in *A. muricata*, with the alkaloid being the second most predominant compound after acetogenin [14].

The comparative antimicrobial activity between the various parts of *A. muricata* and the standard tetracycline revealed that the extract was active or potent enough as the positive control (tetracycline). The ethanolic extract of the leaf was also comparable to tetracycline, and this agrees with a similar study where a methanolic extract of the leaf showed significant antibacterial efficacy and could compete with the standard, streptomycin [26]. However, the difference in this similar report may be attributed to the location of the plant, part of the plant used for the extraction, and the extraction method as these had been reported by Inbathamizh and Padmini [27] and Khattak [28] as some of the factors accounted for the variations.

Methicillin-resistant *Staphylococcus aureus* (MRSA) infection is caused by a bacterium that is resistant to many of the antibiotics used to treat ordinary Staphylococcus infections [29]; hence, the ability of the extract of *A. muricata* to demonstrate antimicrobial activity against MRSA is quite impressive. The high MIC exhibited by the root extract of *A. muricata* (Table 6) suggests a decreased level of antimicrobial agents against the test organism. The combined effect of the various extracts of the plant with the standard antibiotics (streptomycin and tetracycline) showed some increase in the antimicrobial activity, and this could be attributed to the synergistic effect exhibited by their constituents.

The resistance modulation study was carried out by combining the MICs of the standard antimicrobials with subinhibitory concentrations of the various extracts of *A. muricata*, where the subinhibitory concentration is 1/8 of the MIC of the extract, which did not hinder the growth of microorganisms. Reduction in the MIC of the standard antimicrobials in the presence of *A. muricata* extract implies potentiation of activity, whereas an increase in MIC suggested antagonism in the activities of the antimicrobial agents against the test organisms. Four of the aqueous extracts of *A. muricata* antagonized the activity of ampicillin against the tested MRSA by a factor of 0.5 folds (Table 7). The antagonism of the activity of ampicillin may be attributed to the relation between the phytochemicals in the extract and the antibiotics. The phytoconstituents then counter chemically with antibiotics, and this may result in loss of activity [30, 31]. From the results, the combination of the aqueous extracts of the stem, root, seed, and peel with ampicillin should not be encouraged since these reduced the activity of the antibiotic.

Interestingly, due to the interaction of the various extracts with streptomycin and tetracycline, the activities of these two drugs were significantly increased against the MRSA strain used in the range of folds within 1–32 with the aqueous root extract recording the highest synergistic effect and ethanol seed extract with the least effect. This shows that the extracts contain phytochemicals that have either synergistic or inhibitory effects when combined with antibiotics. This enhances the blockade of tetracycline efflux pump activity or modification of the binding site and the biofilm inhibition recorded, which have been identified as the main mechanism of bacteria resistance to antibiotics including tetracyclines. Again, this may probably be due to the extracts' ability to reduce efflux pump and biofilm activity, increasing drug uptake and accumulation which have been identified as the main mechanism of bacteria resistance [31, 32]. Hence, regarding this study, the underlined outcomes show the need to apply caution regarding the indiscriminate combination of herbal medicines with antibiotics to avoid bacterial resistance in most cases but to encourage correct combinations under research in helping to eradicate this menace.

## 5. Conclusion

The results of the present study have shown that the various extracts of *A. muricata* significantly potentiated the efficacy of streptomycin and tetracycline against MRSA. The aqueous root extract recorded the highest synergistic effect and ethanol seed extract with the least effect. It was observed that the combination of the aqueous extracts of the stem, root, seed, and peel with ampicillin should not be encouraged since these reduced the activity of the antibiotic. It is, therefore, recommended that further studies could be carried out to identify the phytoconstituents responsible for the observed potentiation or antagonism.

## Figures and Tables

**Table 1:** List of collected *Annona muricata* plant parts and their voucher numbers.

Plant part used	Voucher specimen number	Location	Geographical location
Leaf	UHAS/ITAM/2021/L002	Woe-Ho, Volta Region	Latitude 6.58096 Longitude 0.46937
Stem bark	UHAS/ITAM/2021/SB001		
Seed	UHAS/ITAM/2021/S001		
Peel	UHAS/ITAM/2021/P001		
Root	UHAS/ITAM/2021/R001		

**Table 2:** Phytochemical screening tests of various extracts of *A. muricata*.

Phytochemical	Test	Observation
Alkaloid	Wagner's reagent (I_2_/KI) was used. A minute amount of extract was dissolved in dilute HCl and filtered. Few drops of Wagner's reagent (I_2_/KI) were added to about 2 mL of the filtrate.	The formation of brownish/red precipitate confirmed the presence of alkaloid.
Flavonoids	Sulphuric acid (H_2_SO_4_) test was done by treating a fraction of the extract with concentrated H_2_SO_4_.	The formation of orange colour confirmed the presence of flavonoids.
Steroids	Liebermann-Buchard test was used. Four milligrams of the extracts was treated with 0.5 mL of acetic anhydride and 0.5 mL of acetic acid. Concentrated H_2_SO_4_ was slowly added.	The surfacing of a reddish/brown colour designating the presence of steroids.
Terpenoids	Liebermann-Buchard test was used. Four milligrams of the extracts was treated with 0.5 mL of acetic anhydride and 0.5 mL of acetic acid. Concentrated H_2_SO_4_ was slowly added.	The appearance of a blue-green colour proving the presence of terpenoids.
Saponins	Exactly 0.5 g of the plant extract was dissolved in 2.5 mL of distilled water. The mixture was shaken vigorously.	The presence of foam indicated the presence of saponins.
Tannins	An exact amount of 0.5 g of the extract was boiled in 20 mL of distilled water and filtered afterwards. Few drops of 0.1% of FeCl_3_ were added.	The presence of brownish-green, brownish-black, or blue-black colour confirmed the presence of tannins.
Glycosides	Benedict's test was used for the detection of glycosides. Precisely, 0.5 g of plant extract was dissolved in 5 mL of distilled water. Exactly 2 mL of benedict's solution was heated, and 8 drops of the dissolved sample were added and allowed to boil for 5 min.	The appearance of a brick red precipitate confirmed the presence of glycosides.

**Table 3:** Phytochemical contents of the various extracts of *A. muricata*.

Parameters	Seed		Root		Leaves		Peels		Stem	
	Aq	Eth	Aq	Eth	Aq	Eth	Aq	Eth	Aq	Eth
Saponins	−	+	+	+	+	+	+	+++	+	++
Alkaloids	+	+	+	+	−	+	−	−	−	+
Terpenoids	−	+	+	++	+	++	++	+++	+	++
Phenols	−	+	+	+	++	+++	+	+	+	++
Tannins	−	+	+	+	++	+++	+	++	+	++
Flavonoids	+	−	+	−	−	+	+	−	+	++
Steroids	−	−	+	−	−	−	−	−	−	+

Aq = aqueous; Eth = ethanolic; + = less concentrated; ++ = moderately concentrated; +++ = highly concentrated; − = negative/not present.

**Table 4:** Results for quantification of biofilm formation of MRSA.

Test microorganism	Optical density of biofilm formation	Interpretation
MRSA	0.727 ± 0.001	+

+ = moderately present.

**Table 5:** Mean zone inhibition of MRSA as exhibited by the plant parts extract.

Plant extracts	Aqueous extract	Ethanolic extract
	Mean zones of growth inhibition of MRSA	
A	15.33 ± 1.20^a^	12.00 ± 0.58^c^
B	17.00 ± 0.00^a^	18.00 ± 0.58^a^
C	9.33 ± 0.67^c^	9.67 ± 0.67^d^
D	9.67 ± 0.67^d^	7.33 ± 1.33^e^
E	11.67 ± 0.33^e^	16.67 ± 1.20^a^
Controls		
Tetracycline	17.00 ± 0.58^a^	17.00 ± 0.58^a^
Water or 20% DMSO	0.00 ± 0.00^b^	0.00 ± 0.00^b^
*Pvalue*	<0.0001	<0.0001

A = root; B = stem; C = seed; D = peel; E = leaf. Values that are in one column with diverse superscript letters are meaningfully different at *P* < 0.05. Values are presented as means ± standard error means.

**Table 6:** Minimum inhibitory concentrations (MICs) and minimum bactericidal concentrations (MBCs) of extracts of the various parts of *A. muricata* and selected antibiotics.

Organism	Minimum inhibitory concentrations of various extracts of *A. muricata* (mg/mL)																			
	ARE		ERE		ASE		ESE		ALE		ELE		ASEE		ESEE		APE		EPE	
MRSA *(NCTC* 29212)	MIC	MBC	MIC	MBC	MIC	MBC	MIC	MBC	MIC	MBC	MIC	MBC	MIC	MBC	MIC	MBC	MIC	MBC	MIC	MBC
	1.0	4.0	0.25	1.0	4.0	8.0	4.0	8.0	1.0	4.0	1.0	4.0	4.0	16.0	4.0	8.0	4.0	8.0	1.0	4.0
	Minimum inhibitory concentrations of the various antibiotics used (µg/mL)																			
	Ampicillin								Streptomycin							Tetracycline				
	32								64							32				

ARE = aqueous root extract, ERE = ethanolic root extract, ASE = aqueous stem extract, ESE = ethanolic stem extract, ASEE = aqueous seed extract, ESEE = ethanolic seed extract, APE = aqueous peel extract, EPE = ethanolic peel extract, ALE = aqueous leaf extract, and ELE = ethanolic leaf extract.

**Table 7:** Modulatory activity of extracts of various parts of *A. muricata* on some selected antibiotics against MRSA.

Organisms	MICs µg/mL of antibiotics + aqueous/ethanolic extracts of *Annona muricata* (0.5 *µ*g/mL) against MRSA														
	Aqueous extracts														
	Ampicillin														
	Root			Stem			Leaf			Seed			Peel		
	A	B	MF/IMA	A	B	MF/IMA	A	B	MF/IMA	A	B	MF/IMA	A	B	MF/IMA
Methicillin-resistant *Staphylococcus aureus* (NCTC 12493)	32.0	64.0	0.5 (−)	32.0	64.0	0.5 (−)	32.0	32.0	1.0 (=)	32.0	64.0	0.5 (−)	32.0	64.0	0.5 (−)
	Tetracycline														
	32.0	8.0	4.0 (+)	32.0	16.0	2.0 (+)	32.0	16.0	2.0 (+)	32.0	16.0	2.0 (+)	32.0	16.0	2.0 (+)
	Streptomycin														
	64.0	2.0	32.0 (+)	64.0	4.0	16.0 (+)	64.0	4.0	16.0 (+)	64.0	16.0	4.0 (+)	64.0	8.0	8.0 (+)
	Ethanolic extracts														
	Ampicillin														
	64.0	32.0	2.0 (+)	64.0	64.0	1.0 (=)	64.0	8.0	8.0 (+)	64.0	64.0	1.0 (=)	64.0	16.0	4.0 (+)
	Tetracycline														
	32.0	16.0	2.0 (+)	32.0	16.0	2.0 (+)	32.0	16.0	2.0 (+)	32.0	16.0	2.0 (+)	32.0	8.0	4.0 (+)
	Streptomycin														
	64.0	16.0	4.0 (+)	64.0	8.0	8.0 (+)	64.0	4.0	16 (+)	64.0	64.0	1.0 (=)	64.0	8.0	8.0 (+)

IMA = interpretation of modulatory activity; MF = modulatory factor; (−) = antagonism; (+) = potentiation; (=) = intermediate; subinhibitory concentration of extract: 0.5 mg/mL, A: MIC of antibiotic alone (*μ*g/mL); B: MIC of antibiotics in the presence of subinhibitory concentration of the extract (*μ*g/mL).

## Data Availability

All the data are available within the manuscript.
